# Supplementary material for: Functional cortical localization of tongue movements using corticokinematic coherence with a deep learning-assisted motion capture system
Source: Sci Rep. 2022 Jan 10;12:388. doi: 10.1038/s41598-021-04469-0 (PMC8748830; doi:10.1038/s41598-021-04469-0)
Supplement: Supplementary file 1 — Supplementary Information. [file 41598_2021_4469_MOESM1_ESM.docx]

**Supplementary Figure 1.**


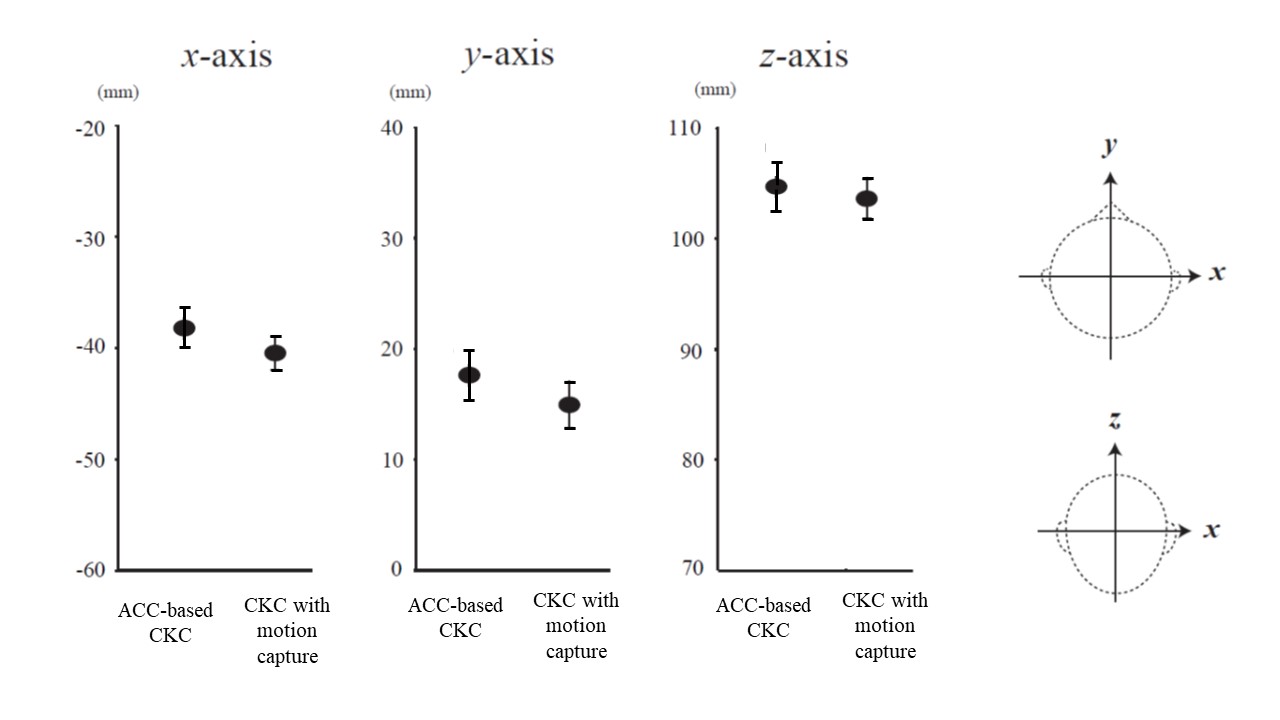


The ECD locations of the right finger over the left hemisphere along each axis (*x*-, *y*-, and *z*-axes) were analyzed between the conventional ACC-based CKC and CKC with deep learning-assisted motion capture using paired *t*-tests with Bonferroni correction.

The corrected *p* value with Bonferroni correction was set to *p* < 0.0167 (0.05/3). The *x*-axis intersected the preauricular points from left to right; the *y*-axis intersected the nasion; the *z*-axis was perpendicular to the plane determined by the *x*- and *y*-axes. The data are expressed as the mean ± SEM. The statistical significance level was set to *p* < 0.05.

The results of the paired *t*-test implied that the locations of the ECDs of the right finger were not difference between the ACC-based CKC and CKC with deep learning-assisted motion capture in all axes. The mean difference between the ACC-based CKC and CKC with deep learning-assisted motion capture were 2.76 mm (*x*-axis), 2.86 mm (*y*-axis), and 1.13 mm (*z*-axis), respectively.

**Supplementary Table 1.**

|  | Peak frequency (Hz) | | | | | |  | CKC value | | |
| --- | --- | --- | --- | --- | --- | --- | --- | --- | --- | --- |
|  | Movement signal | | | CKC | | |  |  |  |  |
| Sub | P | V | A | P | V | A |  | P | V | A |
| 1 | 1.8 | 3.3 | 3.3 | 3 | 3 | 3 |  | 0.69 | 0.69 | 0.71 |
| 2 | 3.3 | 3.3 | 3.3 | 7 | 7 | 7 |  | 0.61 | 0.59 | 0.45 |
| 3 | 2.0 | 2.0 | 2.0 | 4.0 | 4.0 | 4.0 |  | 0.44 | 0.44 | 0.45 |
| 4 | 3.8 | 3.8 | 3.8 | 3.3 | 3.3 | 3.3 |  | 0.32 | 0.31 | 0.31 |
| 5 | 2.0 | 2.0 | 2.0 | 3.5 | 3.5 | 3.5 |  | 0.55 | 0.53 | 0.44 |
| 6 | 1.8 | 1.8 | 1.8 | 3.3 | 3.3 | 3.33 |  | 0.49 | 0.41 | 0.45 |
| 7 | 1.8 | 1.8 | 1.8 | 3.8 | 3.8 | 3.8 |  | 0.32 | 0.31 | 0.30 |
| 8 | 2.8 | 2.8 | 2.8 | 5.5 | 5.5 | 5.5 |  | 0.34 | 0.34 | 0.34 |
| 9 | 2.0 | 2.0 | 2.0 | 2.0 | 2.0 | 2.0 |  | 0.47 | 0.43 | 0.44 |
| 10 | 2.0 | 2.0 | 2.0 | 2.0 | 2.0 | 2.0 |  | 0.55 | 0.56 | 0.56 |
| 11 | 2.5 | 2.5 | 2.5 | 2.5 | 2.5 | 2.5 |  | 0.26 | 0.28 | 0.29 |
| 12 | 2.0 | 2.0 | 2.0 | 2.0 | 2.0 | 2.0 |  | 0.19 | 0.20 | 0.23 |
| Ave | 2.44 | 2.44 | 2.44 | 3.49 | 3.49 | 3.49 |  | 0.433 | 0.424 | 0.413 |
| SEM | 0.20 | 0.20 | 0.20 | 0.43 | 0.43 | 0.43 |  | 0.043 | 0.042 | 0.038 |

P: Position; V: Velocity; A: Acceleration

To assess the validation of the corticokinematic coherence (CKC) with motion capture system using position data, we compared the peak frequencies of power spectrum for motion signals and CKC among approaches using position, velocity and acceleration in the right finger conditions. We also compared the CKC value among approaches using position, velocity and acceleration. The power of motion signals and CKC shows the same peak frequency bands among position, velocity and acceleration, respectively, in all of the subjects. Moreover, as the CKC value obtained from the position (mean: 0.433) reached a similarity of 100.21% and 100.46% when compared with the CKC value from the velocity (mean: 0.424) and acceleration (mean: 0.414), the CKC with capture motion system using position data was found to be a reliable and robust method.

Supplementary Video 1.

Sample video of pose estimation of the tongue during the tongue movement task. The solid blue circles were identified using the learning program with DeepLabCut. The movie is slowed to a quarter of the real-time speed.

Supplementary Video 2.

Movement task of the fingers in the bilateral finger condition. The solid blue (right finger) and red (left finger) circles were identified using the learning program with DeepLabCut. The movie is slowed to a quarter of the real-time speed.
